# Assessing engagement in people living with dementia: A systematised review of existing measures

**DOI:** 10.1002/alz70858_104847

**Published:** 2025-12-26

**Authors:** Kandianos Emmanouil Sakalidis, Martin Ackah, Mohammed Khudair, Arlene Astell

**Affiliations:** ^1^ Northumbria University, Newcastle upon Tyne, United Kingdom; ^2^ Cardiff University, Cardiff, United Kingdom

## Abstract

**Background:**

Engagement can foster greater social inclusion and improve the quality of life of people living with dementia (1). However, different definitions and interventions for studying and increasing engagement exist in the literature (2,3). In an effort to consolidate current understanding of engagement, this review aimed to identify constructs and applications of existing measures for assessing engagement in people living with dementia.

**Method:**

A systematized search strategy was developed and performed in Scopus, Web of Science, CINAHL, and Medline. Original articles containing engagement measures for people living with dementia were included. Following the search, the tittles, abstracts, and full texts were independently evaluated by three reviewers, identifying 21 different studies and measures of engagement.

**Result:**

Diverse terminology for engagement was found across the included studies used. Nineteen of the 21 measures were observational, either real‐time or video analysis. The majority (19 measures) focused solely on behavioral, i.e. observable, indicators of engagement of people living with dementia. Most common behavioral indicators were emotion and affect, verbal engagement, body movements, gestures, and eye gaze. Across measures, engagement was not an on/off state but occurred along a spectrum, from active to passive engagement (or disengagement). Studies were primarily conducted in institutional settings including hospitals, nursing and long‐term care facilities, and were used to assess engagement in a wide range of activities, such as interactions with robots or gardening.

**Conclusion:**

This review revealed the complexity of conceptualizing and assessing engagement by people living with dementia. Most existing measures focus on observable behavioral indicators of engagement and are tailored to specific activities or interactions. The large number of measures developed for use in narrow contexts, highlights a need for more generalizable tools. The findings also suggest that to improve the quality of life of people living with dementia, particularly those in long‐term care settings, more attention is required on how the environment impacts opportunities for engagement, including social engagement.

